# piRNAQuest V.2: an updated resource for searching through the piRNAome of multiple species

**DOI:** 10.1080/15476286.2021.2010960

**Published:** 2021-12-29

**Authors:** Byapti Ghosh, Arijita Sarkar, Sudip Mondal, Namrata Bhattacharya, Sunirmal Khatua, Zhumur Ghosh

**Affiliations:** a Division of Bioinformatics, Bose Institute, Kolkata, India; b Present Affiliation: Department of Orthopaedic Surgery, Keck School of Medicine, University of Southern California, Los Angeles, CA, USA; c Department of Computer Science and Engineering, University of Calcutta, Kolkata, India; d Department of Computer Science and Engineering, Indraprastha Institute of Information Technology, Delhi, India

**Keywords:** PIWI interacting RNAs, piRNA cluster, ping-pong piRNAs, piRNA target, piRNA profile

## Abstract

PIWI interacting RNAs (piRNAs) have emerged as important gene regulators in recent times. Since the release of our first version of piRNAQuest in 2014, lots of novel piRNAs have been annotated in different species other than human, mouse and rat. Such new developments in piRNA research have led us to develop an updated database piRNAQuest V.2. It consists of 92,77,689 piRNA entries for 25 new species of different phylum along with human, mouse and rat. Besides providing primary piRNA features which include their genomic location, with further information on piRNAs overlapping with repeat elements, pseudogenes and syntenic regions, etc., the novel features of this version includes (i) density based cluster prediction, (ii) piRNA expression profile across various healthy and disease systems and (iii) piRNA target prediction. The concept of density-based piRNA cluster identification is robust as it does not consider parametric distribution in its model. The piRNA expression profile for 21 disease systems including cancer have been hosted in addition to 32 tissue specific piRNA expression profile for various species. Further, the piRNA target prediction section includes both predicted and curated piRNA targets within eight disease systems and developmental stages of mouse testis. Further, users can visualize the piRNA-target duplex structure and the ping-pong signature pattern for all the ping-pong piRNA partners in different species. Overall, piRNAQuest V.2 is an updated user-friendly database which will serve as a useful resource to survey, search and retrieve information on piRNAs for multiple species. This freely accessible database is available at http://dibresources.jcbose.ac.in/zhumur/pirnaquest2.

## Introduction

PIWI interacting RNAs (piRNAs) belong to a broad group of endogenous small non-coding RNAs(ncRNAs) [1], which typically ranges in length from 25 to 33 nucleotides (nts). In mammals, these ncRNAs were first reported in mouse testes [2–5]. They act as guide for PIWI proteins, which belongs to Argonaute protein family and exhibit slicer activity [6–9]. Unlike other small ncRNAs, i.e. miRNAs and siRNAs, piRNAs are biogenised from both primary processing pathway as well as the amplifying ping-pong mechanism [10] from single stranded precursor molecules [11] via Dicer independent pathway [12]. The primary piRNAs originate from individual genomic loci that are commonly known as piRNA clusters [10]. In most cases, the germline clusters generate piRNAs from both strands (known as dual-strand clusters), whereas flamenco clusters of *Drosophila* follicle cells and murine pachytene piRNA clusters generate piRNAs from only a single DNA strand (uni-strand clusters) [13]. In the ping-pong cycle, generation of sense secondary piRNAs is initiated by the antisense primary piRNAs which in turn produces secondary antisense piRNAs and the amplifying loop continues [7,10].

Although studies on fish, flies and mammals have shown a conserved association of piRNAs with PIWI proteins [2,10,11], the length variation of piRNAs have been observed with evolving sequencing technologies between different species. In general, piRNAs in mammals can be categorized into two subclasses called pachytene (29–33 nts) and pre-pachytene (26–28 nts) [14], whereas piRNAs in *Caenorhabditis elegans* are named as 21 U-RNA owing to its bias for length of 21 nts. Though the piRNAs are best seen in germ cells, several studies have shown piRNA expression in brain, kidney, lung, liver, stomach, testis and ovary [15–18] as well as in different cancers [19].

To maintain genome integrity in germ cell lineages, highly expressed PIWI proteins in germ and stem cells [9] take part in controlling transposon activity as a defensive mechanism [20]. Studies showed that, mutation in MIWI which is a PIWI homolog in mouse leads to male infertility as well as over expression of retrotransposon transcripts [21]. Similar observation has been reported in case of flies [12]. In association with piRNA forming piRNA-induced silencing complexes (piRISCs), PIWI-piRNA pathway silences transposons via complementary base-pair recognition between piRNA and transposon followed by endonucleolytic cleaving of the target [22,23].

Existing databases like piRNABank [24], piRBase [25], piRNAdb [https://www.pirnadb.org], piRTarBase [26] and piRNA Cluster Database [27] provide information on piRNAs for multiple species. Among these, piRBase is a manually curated database which hosts piRNA information on multiple species and some disease systems. ‘piRNA cluster database’ is a dedicated database for piRNA clusters where the clusters are predicted using proTRAC [28]. piRDisease V1.0 [29] hosts piRNA records for different diseases but is not currently accessible. Despite such extensive work on piRNAs, there still remain several unexplored areas, such as their association with long noncoding RNAs (lncRNAs) or the presence of any genomic elements within their loci which can influence their function. We published the first version of piRNAQuest to probe deep into these lesser explored domains of piRNAome. It hosted piRNA information for three species, viz. human, rat and mouse [30].

Though various computational tools have characterized novel piRNAs [31,32] but their function remains unclear. Hence, it is important to identify potential piRNA targets and disease-related piRNAs. Further, both predicted and validated piRNA targets including mRNAs and lncRNAs are not properly curated in any of the existing databases. Moreover, identifying piRNA clusters which are hotspots of piRNA biogenesis is another big challenge in piRNA research.

In this work, we present piRNAQuest V.2, which is an extended version of piRNAQuest. This new version includes the following additional features: (i) extensive analysis on 25 new species in addition to human, mouse and rat of the previous version, (ii) density-based clustering approach [33] to identify the ‘hotspots of piRNA expression’, popularly known as ‘piRNA clusters’. Since piRNA distribution varies with genomic locations in different species, identifying piRNA clusters based on their density in genome can provide new impetus to get biologically relevant clusters, (iii) tissue specific expression of piRNAs among different species, and (iv) expression of piRNAs in different disease systems with an emphasis on different types of cancers. Emerging evidences suggest that piRNAs have important roles in disease progression and diagnosis [34–39]. Thus, the efficacy and potential mechanism of action of a piRNA in cancer relies on its expression in various tissues and disease systems which correlate with disease progression, (v) piRNA target prediction within both mRNAs and lncRNAs that would further help to identify the key players contributing towards disease development.

In addition to these extensive features, we have updated another section of the database, viz. ‘Tools’, where users will be able to predict piRNA clusters using customized parameters, check ping-pong pattern overlap in their sequences and predict piRNA targets using miRanda [40].

Overall, piRNAQuestV.2 is a user friendly database for multi-species piRNA survey, search and retrieval. piRNA expression within normal tissues and cancer as well as the information about piRNA targets will serve as a valuable resource for piRNA researchers. The database is freely accessible at http://dibresources.jcbose.ac.in/zhumur/pirnaquest2.

## Results

**Figure 1.**
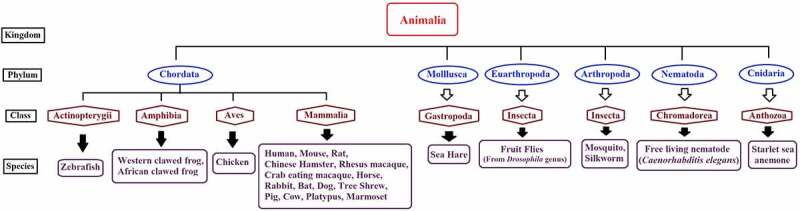
Taxonomical representation of species included in piRNAQuest V.2.

piRNAQuest V.2 (an updated version of piRNAQuest) hosts information on 92,77,689 piRNAs corresponding to 28 species (consisting of 25 new species in addition to human, mouse and rat) which are from different phylum ranging from nematode to chordate (Figure 1). Apart from the coverage of species, this new version has included several additional features which add to the significance of this database as compared to other piRNA database. The set of updated features of this new version compared to the old version has been put up in Table 1. We have also put up feature wise comparison of piRNAQuest V.2 with other piRNA database (Supplementary File S1).

**Table 1.** Comparison of features between piRNAQuest V.2 and piRNAQuest

Database content	piRNAQuest	piRNAQuest V.2
Number of species	3	28
piRNA entries	9,98,585	92,77,689
Chromosomal distribution	Yes	Yes
Association with gene	Yes	Yes
Association with pseudogene	Yes	Yes
Association with repeat elements	Yes	Yes
Cluster information	Yes (Lau et al. method), for 3 species	Yes (Density based clustering approach), for 19 species
Association of clusters with genomic regions	Yes	Yes
Syntenic piRNA clusters	Yes	Yes
Ping-pong piRNAs	Yes	Yes
*Ping-pong pattern Visualization*	No	Yes
Tissue specific expression	Yes (Tissue type – 6, No. of Samples – 9)	Yes (Noraml Tissue type – 32, No. of Samples – 243)
*piRNA disease association*	No	Yes (16 types of cancer, 2 neurodegenerative diseases amd 3 other diseases)
*Graphical representation of expression*	No	Yes (For 32 normal tissue types and 16 types of cancer, 2 neurodegenerative diseases amd 3 other diseases)
*Predicted piRNA – mRNA target pairs*	No	Yes (For seven types of cancer, asthenozoospermia and mouse testis)
*Predicted piRNA targets within lncRNAs*	No	Yes (For seven types of cancers, asthenozoospermia and mouse testis)
*piRNA target genes (literature curated)*	No	Yes (for Human, Mouse and *C. elegans*)
*Target prediction tool*	No	Yes
*Ping-pong overlap prediction tool*	No	Yes

Common names are used for the species with their respective Kingdom, Phylum and Class.

Among 28 species, 9 species (viz. Chinese hamster, Sea hare, Tree Shrew, Brown Bat, Silkworm, Mosquito, *Drosophila virilis, Drosophila erecta* and Starlet sea anemone) has not been annotated yet. Hence, genomic localization and related features could only be provided for the rest of the 19 species. Among rest of the 9 species, we have been able to identify repeat-associated piRNAs for 4 species (viz. Chinese hamster, Tree Shrew, *Drosophila virilis* and *Drosophila erecta*), as their repeat annotations were available from UCSC [41] and this information can be visualized in graphical format from the ‘Statistics’ submenu under ‘Help Menu’ of the database.

### Genomic localization based distribution of piRNAs

piRNAQuest V.2 hosts multispecies piRNA information where there is a remarkable increase in the number of piRNA entries compared to that in the previous version. Among the 28 species, distribution of piRNAs across different chromosomes has been mapped only for 19 species (as mentioned above) (Supplementary Figure SF1 and SF2). Interestingly, chromosome 15 in human contains the maximum number of piRNAs which is similar to our observation reported in the earlier version of piRNAquest [30]. In this connection, it is important to note that chromosome 15 in human has been reported to harbour large number of low copy repeats popularly known as duplicons [42] which facilitate nonhomologous recombination events [42] that leads to genome instability [43]. Presence of maximum number of piRNAs in the same chromosome might be to overcome such adverse situation of genome instability, as piRNAs are known to play significant role towards maintaining genome integrity [12].

Further, chromosome 7 and 1 of mouse and rat respectively harbours the maximum number of piRNAs. Among the newly added species, chromosome IV of *Caenorhabditis elegans* (which has also been reported earlier [44]) and Chromosome 2R of *Drosophila melanogaster* contains maximum number of piRNAs.

**Figure 2.**
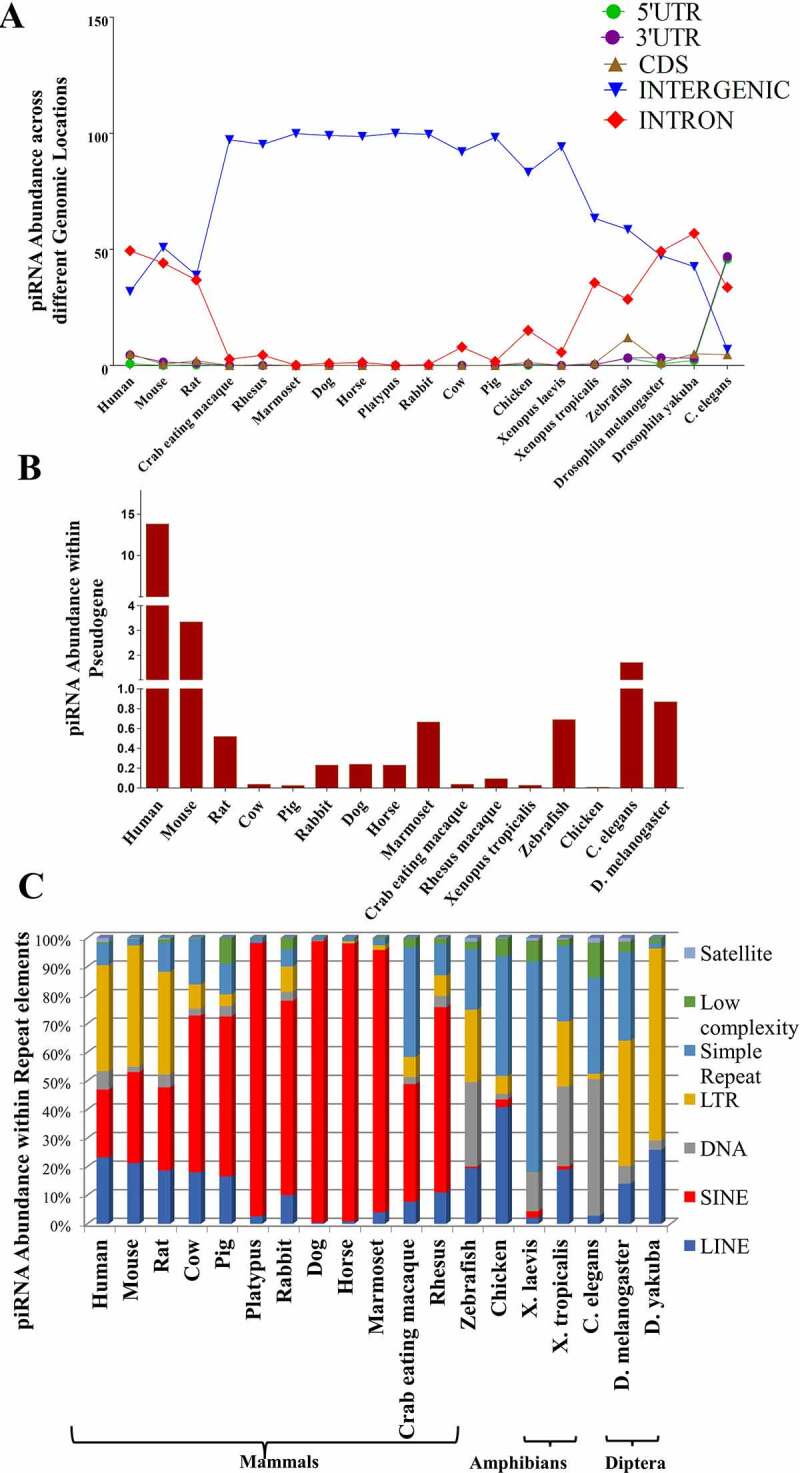
Distribution of multispecies piRNAs: (a) across different genomic locations, (b) within pseudogenes and (c) within repeat family.

Abundance of piRNAs in intergenic regions is mostly predominant as compared to that in intronic region for most of the species (Figure 2(a)). One of the significant functions of these intergenic piRNAs is their involvement in early embryonic development [45]. Further, it has been reported that intergenic regions harbour lncRNA loci [46]. Hence, we have checked for the presence of lncRNA loci overlapping with piRNA clusters which consists of intergenic piRNAs (results shown later under the section ‘piRNA clusters overlapping with lncRNAs’). On the contrary, piRNA abundance in the 3’ UTR, 5’ UTR and CDS region is less, except in zebrafish (having high piRNA abundance in CDS region) and *C. elegans* (having high piRNA abundance in 3^/^ UTR and 5^/^ UTR regions) (Figure 2(a)).

Abbreviations used: UTR- untranslated region, CDS – coding DNA sequence, LTR – Long-terminal repeat, LINE – Long interspersed nuclear elements, SINE – Short interspersed nuclear elements.

Further, it has been found that pseudogenes regulate its counter gene stability via small RNA mediated silencing [47]. Recently, it has been reported that pachytene piRNAs from pseudogenes directly regulate its parent genes [48]. This motivated us to check the presence of piRNAs within pseudogenes for 16 species whose pseudogene information is available [49]. We obtained significant overlap between piRNAs and pseudogenes in several species (Figure 2(b)). Interestingly, maximum overlap of piRNAs with pseudogenes has been observed in human. Recently, pseudogene derived piRNAs have been found in mature human sperm cells which indicate their role in regulating expression of their parent gene in male germline cells [50].

### Distribution of piRNA within repeat regions

piRNAs have been reported to have originated from the repetitive regions and they silence transposons in insects and mice [10,51,52] regulating global gene expression during embryonic development [52]. The piRNA loci for all the 19 species have been mapped to the genomic locations corresponding to seven major categories of repeat elements, viz. LINE, SINE, Simple repeat, DNA, Low complexity, Satellite and LTR (Figure 2(c)). Vandewege et al. [53] reported a strong piRNA response in mammals like dog and horse. These piRNAs are harboured within the SINE repeat regions which are mostly abundant within these species. In our database, we have also reported the enrichment of SINE repeat associated piRNA loci for 12 mammalian species. In addition to this, several human, mouse and rat piRNA loci overlap with LTR repeat family. On the contrary, amphibian piRNAs show a tendency to overlap with DNA and Simple repeat family. Petersen et al. [54] has reported the abundance of LTR repeats within those genomic loci corresponding to the transposable elements present in Diptera (a particular order of insect class). Our study also reveals similar observation in case of the order Diptera, where piRNA enriched regions corresponding to this order overlap with LTR repeat family. Presence of such repeat regions within piRNA loci can have important implications as is shown by Halbach et al. [52]. Here, it has been reported that satellite repeats modulate global gene expression via piRNA-mediated gene silencing which is important for embryonic development of Aedes.

### Biogenesis of piRNAs – the piRNA clusters and ping-pong amplification

piRNA clusters are also known as the hotspots of piRNA biogenesis. Initially, in the first version of piRNAQuest, the method described by Lau et al. [55] was followed to identify the piRNA clusters within a chromosome. Here a fixed window length of 20 kilobases (kb) was used to identify the clusters. Later in 2016, Rosenkranz reported that the piRNA clusters are not equally distributed across the chromosomes and is not even related to the length of the chromosome [27]. As the piRNA read distribution varies across the genome corresponding to different chromosomes, one should not fix the window size for detecting piRNA cluster. Hence, in this new version of our database, piRNAQuest V.2, we have adopted density based clustering approach [33] to identify the piRNA clusters (Supplementary Figures SF3 and SF4) which was found to be effective to recognize clusters successfully in chicken germ cell [56].

We obtained maximum no. of clusters in chromosome 15 and chromosome IV for human (Supplementary Figure SF3) and *C. elegans* respectively (Supplementary Figure SF4). We also found the same for human previously. In *C. elegans*, it is reported that maximum clusters lie within chromosome IV [44]. Though the function is still unknown, it has been found that among the sex determining chromosomes, ‘X’ chromosomal piRNAs mainly originate from clusters compared to the ‘Y’ chromosomal piRNAs [27]. Our analyses also have revealed more piRNA clusters in ‘X’ chromosomes than that in ‘Y’ chromosome of human, mouse and rat.

*piRNA clusters overlapping with lncRNAs*: We have studied the distribution of piRNA clusters within the lncRNA loci obtained from LncRBase V.2 [57]. As mentioned earlier, in the first point of this result section, we observed a significant overlap of piRNA clusters with the intergenic lncRNAs (Supplementary Figure SF5A) which are transcribed from in between two gene loci. This goes in line with previous reports [58]. In addition, we looked at the overlap of piRNA clusters with repeat regions (Supplementary Figure SF5B) and found similar observation as that obtained from the distribution of piRNAs in repeat elements (as shown in Figure 2(c)).

*Motifs within piRNA clusters*: Characteristic motifs have been identified for each of the clusters. These highly conserved motifs within the piRNA clusters may provide us information on possible common piRNA binding sites within its target gene. piRNAs from a cluster generated from coding gene regions can also regulate its ‘host’ gene expression [59]. A significant % of total piRNA clusters have been found to be overlapping with coding regions in many species (Supplementary Figure SF5C).

piRNAs are also generated via secondary biogenesis or the ping-pong amplification loop. Studies on fly have shown that somatic piRNAs generally do not show ping-pong pattern, suggesting that the ping-pong loop may work mainly in germline cells [60,61]. The distribution of ping-pong piRNAs among the different chromosomes was determined. Chromosome 15 in human shows predominance for ping-pong piRNAs and has also been reported very recently by Ray and Pandey [62]. More than 50% of the ping-pong piRNAs in human are found to overlap with protein-coding genes which indicates towards piRNA-dependent gene regulation [63]. Further, less than 10% of these piRNAs are found to overlap with repeat elements among which SINE repeat family is predominant. Previously, Das et al. [64] showed that ping-pong amplification does not occur in nematode, but surprisingly in our analysis, 509 ping-pong piRNAs are found in chromosome IV of *C. elegans* which may instigate the role of ping-pong loop in nematode as well.

### Tissue-specific expression of piRNAs

Initially, piRNAs were observed to be expressed exclusively in germline cells [3]. But gradually they have been identified in somatic cells and the somatic piRNA pathway have been seen to regulate germline transpositions [65]. Hence, we have analysed 243 small RNA sequencing samples for 32 tissue types corresponding to 25 species (Supplementary File S2) in which 13 tissue types are from human. Supplementary Figure SF6 reveals the expression pattern of piRNAs among different tissue types corresponding to all these 25 species. For human, we have found the presence of maximum number of piRNAs in brain followed by colon, testis, spermatozoa which indicates the role of piRNAs not only in the germline cells, but also in other somatic cells. Previously, it was shown that there are piRNA complexes in mouse dendritic spines of brain and knockdown of those piRNAs resulted in lower spine density in the axons [45]. Recent studies also indicate that piRNAs in brain are associated in suppressing retrotransposons. This has a significant role in brain pathology [66]. It has been found that the piRNA length distribution is related to the age of the individual belonging to a particular species, e.g. in Drosophila the length of piRNAs becomes shorter with age [67]. Further, loss of methyltranferase result in piRNA instability and reduction in piRNA length and volume, which ultimately leads to male sterility during spermatogenesis [68]. Interestingly, in our study, we have found the presence of piRNAs, which are around 36 nts in length in human sperm samples, whereas such longer piRNAs have been seen to be expressed very less in any somatic cells.

### Disease specific expression of piRNAs

**Figure 3.**
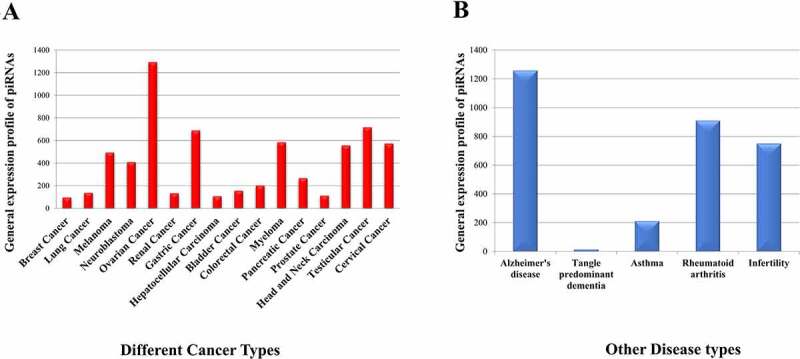
General expression profile of piRNAs in (a) different cancers and (b) other disease systems.

With developments in pathological research, studies have highlighted the importance of piRNAs in disease systems. piRNAs and PIWI proteins are found to be expressed abnormally in several cancer systems that increases their importance as potential novel biomarkers for therapeutic research [19]. Recent evidences suggest that genomic stability of neurons may be disturbed by dysregulation of the piRNA pathways which results in various neurodegenerative disorders [69]. As genes involved in the biogenesis of piRNAs have an essential role in spermatogenesis, mutation in those genes may lead to male infertility [70]. Besides, piRNAs are shown to regulate Th2 cell development by downregulating IL-4, thus inhibiting allergic inflammation and asthma [71] and have specific binding partners in synovial fibroblasts, suggesting its role in inflammatory processes like Rheumatoid Arthritis [72]. Here, we have analysed 211 samples corresponding to 21 disease types (Supplementary File S3) in which 16 types of cancer are present. The distribution of piRNAs (Figure 3(a)) among different cancers shows the higher contribution of piRNAs in germ cell cancers like ovarian and testicular cancer. Here, our observation goes in line with the established role of piRNAs towards maintaining germ cells [73].

Among other diseases (Figure 3(b)), we found the presence of 1274 piRNAs among which hsa_piRNA_425 is highly abundant and hsa_piRNA_28207 is lowly abundant as compared to the abundance of other piRNAs in Alzheimer’s disease. These have been reported previously [36]. The number of piRNAs expressed in asthma and rheumatoid arthritis are 278 and 910 respectively. Another interesting observation in this dataset is regarding the length of the piRNAs. In our study, we have observed the presence of longer piRNAs in sperm sample where maximum length of piRNAs is 32 nts in case of infertile samples indicating the significance of piRNA length towards spermatogenesis [68].

Beside the general expression profile of piRNAs in different diseases, differential expression analysis has been also performed using DESeq [74] to see the differential mode of regulation of piRNAs in seven cancer systems and asthenozoospermia (based on the availability of both test and control datasets). Table 2 shows the number of differentially expressed piRNAs among which the expression of some piRNAs corroborated with that obtained from literature evidences. For example, hsa_piRNA_9871 and hsa_piRNA_27200 are found to be upregulated in breast and lung cancer respectively [75]. This has also been observed in our study. The upregulated piRNAs hsa_piRNA_7806 and hsa_piRNA_31147 promote proliferation and invasiveness in colon [76] and renal cancer [77], respectively, and are also observed to be upregulated in our analysis.

**Table 2.** Differentially expressed piRNAs, genes and lncRNAs in different cancer systems, Asthenozoospermia and different developmental stages of mouse testis

	Differentially expressed piRNAs		Differentially expressed Genes		Differentially expressed lncRNAs					
	Upregulated piRNAs	Downregulated piRNAs	Upregulated genes	Downregulated genes	Upregulated lncRNAs	Downregulated lncRNAs				
*Different Cancer systems*										
**Breast cancer**	253	133	955	1165	1087	615				
**Lung cancer**	269	349	517	513	53	82				
**Ovarian cancer**	208	376	3300	2376	335	1441				
**Renal cancer**	177	724	197	162	85	40				
**Hepatocellular carcinoma**	352	155	116	125	169	171				
**Colon cancer**	366	475	344	361	79	119				
**Prostate cancer**	413	328	2135	2368	504	179				
*Other disease*										
**Asthenozoospermia**	1622	2170	318	133	2957	1062				
*Different developmental stages of mouse testis*										
**10 dpp**	923	439	2173	2429	1445	1442				
**16.5 dpc**	260	143	7281	6498	3209	2430				

### piRNA-target gene interaction

**Figure 4.**
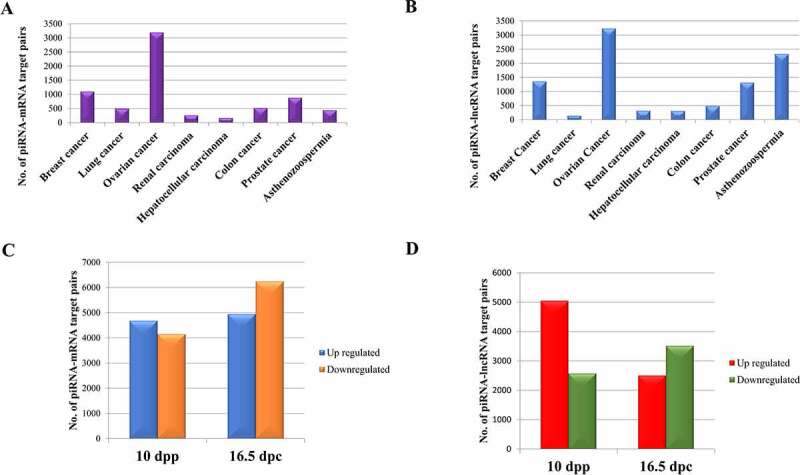
Predicted piRNA targets in (a) protein coding genes and (b) LncRNAs within disease systems; (c) protein coding genes and (d) LncRNAs across different developmental stages of mouse testis.

Beside piRNA mediated cleavage of transposable elements, piRNAs are also known to target mRNAs and lncRNAs and subsequently regulate their expression. The involvement of piRNAs in regulating mRNAs has been studied extensively [36,78,79]. In a way, similar to the slicing of mRNAs, PIWI-piRNA complex can target lncRNAs which has been observed in multiple organisms [80]. It has been reported that a decrease in the expression levels of the target may correspond to an increase in the expression levels of the targeting piRNA, and vice versa [81]. Hence, for precise target prediction, we have screened those piRNAs and mRNAs as well as piRNAs and lncRNAs whose expression are negatively correlated. This has limited our analysis to those cancer datasets where both long and small RNA seq datasets are available. Hence, we have been able to predict piRNA-mRNA and piRNA-lncRNA interaction for 7 cancer systems (viz. lung, breast, renal, hepatocellular, ovarian, prostate and colorectal). The input dataset have been shown in Supplementary File S4 and the differential analysis was performed using the ‘New Tuxedo’ protocol [82]. Sequence based target prediction has been done using miRanda. Tissue and cell line data have been analysed separately. In order to highlight the role of piRNAs in different developmental stages, we have analysed the small RNA data corresponding to different developmental stages of mouse testis viz. 10dpp (days post-partum) and 16.5dpc (days postcoitum) as compared to that of six months old adult mouse testis. Further, piRNAs have been analysed corresponding to another disease system named asthenozoospermia where the sperm motility gets reduced in semen sample. The differentially expressed mRNAs, lncRNAs and piRNAs are mentioned in Table 2. The final set of piRNA-mRNA and piRNA-lncRNA target pairs for 7 cancer types is shown in Figure 4(a,b), respectively. Figure 4(c,d) shows the number of piRNA targets within mRNAs and lncRNAs respectively in two developmental stages of mouse testis. Moreover, we have also curated experimentally validated piRNA–mRNA target pairs for human, mouse and *C. elegans*.

## Discussion

There has been an increase in the number of piRNAs that have been identified in different species as well as in different cells since our first release of piRNAQuest in 2014. Initially, we developed piRNAQuest, with a goal to develop a non-redundant comprehensive catalogue of human, mouse and rat piRNAs so as to provide a better understanding regarding their genomic localization, overlaps with genomic elements and their association with other lncRNAs. Although, initial reports reveal the main functions of piRNAs to be transposon silencing [51] and maintenance of gene integrity mainly in germline cells [9], but later it has been identified in somatic cells as well in many species [10,12]. All these put forward, the increasing importance of its diverse functions not only in transposon silencing but also in gene expression regulation. Hence, we have come up with this new version of piRNAQuest named as piRNAQuest V.2, where we have expanded our study to 25 new species (apart from those included in previous version) covering different phylum or classes. Along with the previous features, piRNAQuest V.2 has focused on several new aspects such as directionality of piRNA cluster, piRNA expression among normal tissues and disease systems and its targets among protein coding genes and lncRNAs. These will open up novel avenues for piRNA research.

Over time, many studies have demonstrated the mechanism of primary biogenesis of piRNAs from piRNA clusters [22,23]. Several protocols have been developed to identify them. However, lack of uniform distribution of piRNAs among the chromosomes lead us to consider the density based clustering approach to identify piRNA clusters. It will help in understanding the distribution of piRNAs throughout the genome and the formation of clusters which are the ‘hotspots’ of piRNAs for primary biogenesis. Besides, secondary biogenesis via ping-pong amplification is also important for generation of piRNAs and its role towards silencing of its target. Emphasizing on this, we checked the ping-pong overlap among the piRNAs and have also provided options to visualize the ping-pong signature within the piRNAs. In human, we have seen the presence of maximum piRNAs in chromosome 15 where the maximum number of piRNA clusters and ping-pong piRNAs are also present.

In addition to this, analysing piRNA expression profile of various normal and disease systems will help us to understand the piRNA-mediated gene regulation in those systems. In this version, we have incorporated the piRNA expression profile of 21 disease systems along with several normal tissue data corresponding to different species. As piRNAs are differentially regulated between disease and normal conditions, a decrease in the expression levels of the target should correspond to an increase in the expression levels of the targeting piRNA, and vice versa. Taking this as an opportunity, to unravel the connection between piRNA expression and disease occurrence, we have predicted probable piRNA targets which may serve as promising biomarkers for early diagnosis and act as therapeutic targets for diseases like cancer. Further, in order to show the involvement of piRNAs in different developmental stages, we have predicted piRNA targets within mRNAs and lncRNAs in different developmental stages of mouse testis.

Overall, the newly added features along with the existing ones will make piRNAQuest V.2 a user friendly, comprehensive database for piRNAs. Our future goal is to update the database regularly with newly annotated piRNAs along with its novel features in order to continue contributing to the growing piRNA knowledgebase.

## Materials and method

### Improved content and new features

#### Input dataset

piRNA entries have been extended to 25 new species in addition to human, rat and mouse. The genome builds, availability of genome annotation and repeat annotation information and the number of piRNAs corresponding to the species has been mentioned in **Supplementary Table ST1**. The genome builds are updated from hg19 to hg38 and rn5.0 to rn6.0 for human and rat respectively. Data are collected in different formats like fasta, gtf and bed from the respective sources. Repeat elements and Refseq annotated 5^/^UTR, 3^/^UTR, exon, intron and CDS information have been downloaded from UCSC [41]. The miRNA information has been downloaded from miRBase 22 release. Annotated piRNA sequences were downloaded in.fasta format from National Centre for Biotechnology Information (NCBI) [83]. Normal tissue and disease specific small and long RNA sequencing data has been obtained from NCBI Gene Expression Omnibus (GEO) [84]. LncRNA information has been retrieved from LncRBase V.2 [57].

#### Data processing and refinement

***Redundancy check and ID assignment***: The procedure of assigning IDs to non redundant piRNA entries is similar to that followed in piRNAQuest [30]. The sequencing data were aligned to respective genome. We further filtered out those reads mapped to other ncRNAs and screened the reads predicted to be piRNAs using our in-house script. Thereafter, non-redundant reads were re-aligned with reference genome for complete alignment with no mismatches and annotated with unique piRNAQuest IDs, i.e. [three letter abbreviation of species name]_piRNA_[number]. The annotation IDs are same for human and mouse as assigned in the previous version. The only difference is in the annotation of rat from the previous one as in the last version it was not annotated as three letter abbreviation of species name. Users can find the previous IDs which are annotated in this version of the database in the ID conversion of Help menu for human, mouse and rat. To study the distribution of piRNAs within genome, we searched for the localization of piRNAs within gene, intergenic regions, intron, CDS, UTR regions, repeat elements and pseudogenes using in-house perl scripts as that followed in the first version.

***Density based piRNA cluster prediction***: Previously used cluster prediction protocol [55] have a disadvantage of considering window size of fixed length for all species and hence does not account for the variation in read distribution among different species. To overcome this discrepancy, we have adopted density based clustering algorithm DBSCAN [33] to develop a python based in-house protocol for identifying piRNA clusters which is based on the read distribution of piRNAs across the genome.

*Clustering parameters*: There are two parameters ‘Eps (or epsilon)’ and ‘MinReads’ which allow us to find candidate clusters. ‘Eps’ is defined as the distance of a read from a neighbourhood point and ‘MinReads’ are the minimum number of reads within ‘eps’ distance. To determine the clustering parameters, inter distance between the annotated piRNAs are calculated by performing k-dist analysis [33]. We calculated the distance between each mapped read and its kth nearest neighbouring read which is referred to as k-dist which is plotted with respect to the its counts. Eventually, a sharp valley has been observed in this ‘count versus distance’ plot until the k-dist follows a uniform distribution. The distance, for a given value of k, after which the graphs follows an asymptotic decrease is termed as the eps i.e. eps represents the distance which repeats itself for maximum number of times and hence has the highest probability of defining the boundaries of a cluster containing at least, the ‘MinReads’ which represents the number of reads within the cluster. After the ‘Eps’ and ‘MinReads’ parameters are set for each chromosome corresponding to each species, clusters are detected from the coordinate file of the annotated piRNAs.

*Cluster score*: In order to calculate piRNA enrichment within each cluster, we have calculated cluster score for each piRNA clusters. This has been calculated as follows:

(1) $$Cluster\;\;score=\;\frac{Total\;\;no\;\;of\;\;piRNAs\;\;in\;\;the\;\;cluster}{Minimum\;\;no\;\;of\;\;piRNAs\;\;needed\;\;to\;\;form\;\;the\;\;cluster\left(kth\;\;value\right)}$$

We also checked for the strand specificity of the clusters based on the directionality of the constituent piRNAs within that cluster. If a cluster contains both sense and antisense piRNAs, it is considered as ‘dual strand cluster’ and if it contains only sense piRNAs or antisense piRNAs, it is considered as ‘uni-strand cluster’.

*Localization of piRNA clusters and characteristic motifs within them*: (i) In-house perl scripts have been used to check for any overlap of piRNA clusters with coding genes or lncRNAs or the repeat regions. (ii) piRNAs show a strong tendency to form clusters in the syntenic regions of genome [3]. We have downloaded the syntenic regions from UCSC [41] and searched for piRNA clusters in the corresponding syntenic regions among different species. (iii) MEME have been used to find the presence of any significant motifs within the piRNA clusters [85].

***Ping-pong pattern within piRNAs***: The secondary mode of piRNA biogenesis, i.e ping pong amplification shows a distinct sequence based feature within the piRNAs, i.e. a 10 nt overlap is found between the antisense and sense piRNAs. An in-house python script has been developed to identify these ping-pong piRNAs and visualize this ping-pong signature pattern.

***piRNA profile in Normal and Disease systems***: We have downloaded small RNA sequencing data for different tissue types from GEO (https://www.ncbi.nlm.nih.gov/geo). A total of 243 samples were analysed for 32 types of normal tissue samples for different species. Along with the normal dataset, we have analysed 211 samples corresponding to 21 types of disease data, which includes 16 different types of cancer data sets. To analyse the expression profile of piRNAs among the normal tissue and disease systems, BLAST [86] and in-house perl scripts were used. Further the expression level of each piRNA found in a sample was normalized by counts per million (CPM) and were further screened based on the z-score [87] lying between −3 and +3. Users will be able to view the expression of 200 most abundant piRNAs in each set. Additionally, we have checked the distribution of each piRNAs among all the normal tissues or disease systems which has been represented graphically to provide better understanding regarding their expression within different systems.

***piRNA Target prediction***: piRNA target pairs have been predicted between up regulated piRNAs and downregulated lncRNAs and mRNAs and vice versa. miRanda [40] has been used for predicting piRNA targets within lncRNAs (sequences obtained from LncRBase V.2 [57]) and 3^/^ UTR region of mRNAs (sequences downloaded from UCSC [41]). The target score and energy threshold are 170 and −20 kcal/mol respectively [88]. In the database, we have linked The Human Protein Atlas [89] and Pathway Commons [90] databases to the targeted genes for further pathway and pathology based analysis. Further, our database hosts several experimentally validated piRNA targets for human, mouse and *C. elegans* which have been manually curated from published reports.

The overall workflow has been outlined in (Supplementary Figure SF7).

### Database execution

**Figure 5.**
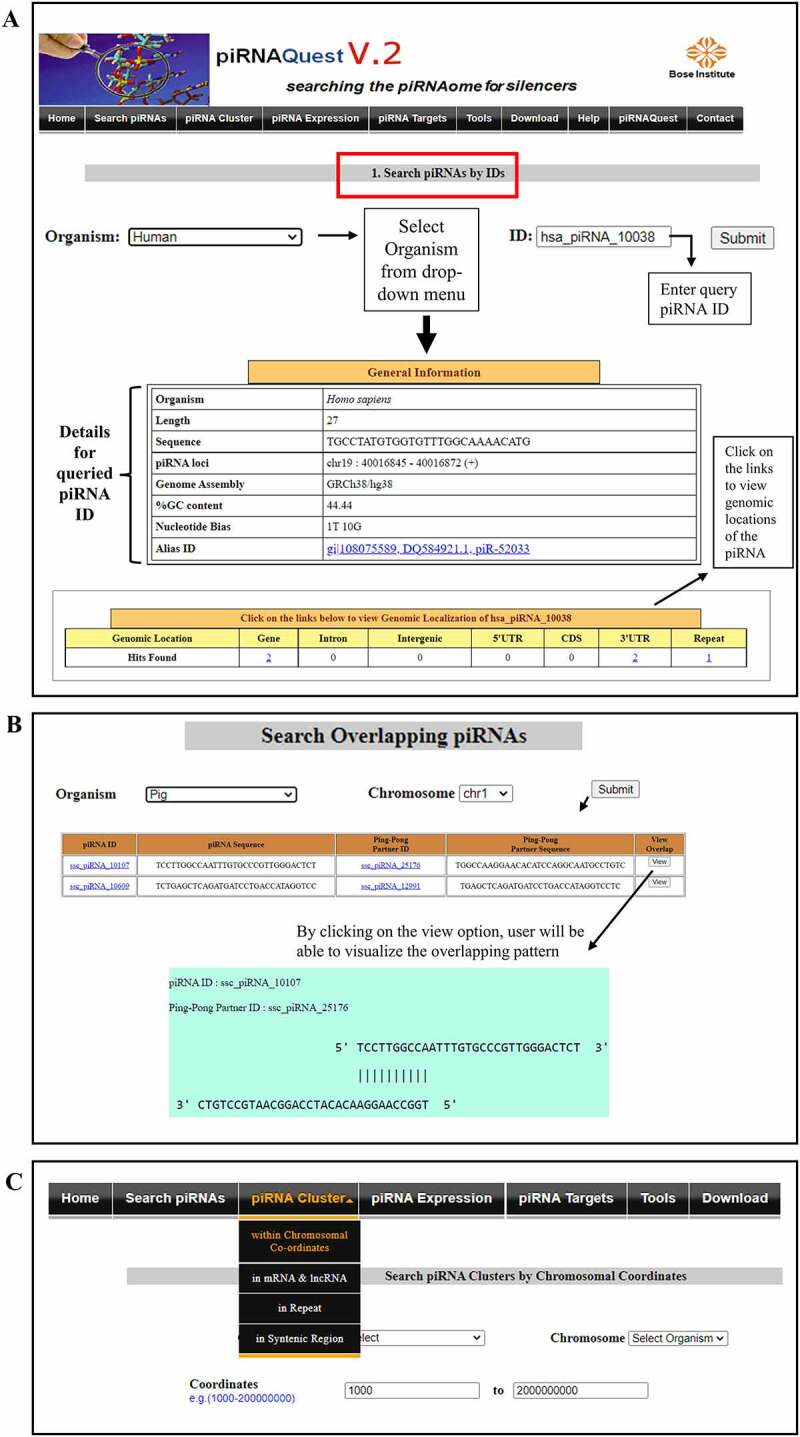
Web interfaces for easy access of piRNAQuest V.2 showing: (a) search options through a piRNA ID and the corresponding result page; (b) search options for pingpong piRNAs and visualizing its pattern; and (c) search options to browse piRNA clusters.

In piRNAQuest V.2, a query is basically processed via simple searching options using user’s desired selection criterion and information are presented on the web interface after retrieving related details from the database. The general information page displays basic information related to the piRNAs and provides options to probe into its further genomic details which is shown in Figure 5(a).

#### Search and output options

(a) The following options are under the ‘**Search piRNAs**’ menu

1. ***Search by Species Name***: Users can browse all piRNAs by selecting a particular species name with the help of previous or next buttons.
2. ***Search by piRNA Accession ID/Chromosomal Co-ordinates***: Users can search by piRNA accession ID for detailed information (piRNA sequence, its length, its NCBI ID (if any), %GC content, piRNA position corresponding to the genome build along with its genomic localization within genes, introns, CDS, 3^/^UTR, 5^/^UTR, intergenic regions, and repetitive elements) of selected species. A piRNA ID has already been provided as an example for each of the species. Using desired chromosomal co-ordinates, users can also get above mentioned information about piRNAs.
3. ***Search piRNAs by Sequence:*** Users can retrieve piRNA information by providing piRNA sequences. The sequence length should be greater than at least 20 nucleotides.
4. ***Search piRNA within Genes:***User can search piRNAs present within Genes by providing a Gene Name corresponding to the selected species. The result page will show the piRNAs whose loci overlappes with this particular gene. User can also search for piRNAs within Genes of a particular species by providing chromosomal coordinates corresponding to that species.
5. ***Search piRNA within repeats:*** Users can search for piRNAs whose loci get mapped within repeats corresponding to genomic locations (viz. 3/UTR, 5/UTR, introns, CDS, intergenic regions) for a particular Repeat Family. Users can also search for repeat-associated piRNAs selecting their desired chromosomal location.
6. ***Search piRNAs with Ping-Pong features:*** User can search for 10nt overlapping piRNAs within a particular chromosome of a particular species by selecting chromosome number corresponding to that species Figure 5(b).

(b) Search ‘**piRNA clusters’**

1. ***Search clusters by chromosomal co-ordinates***: Users can obtain piRNA clusters by submitting a particular chromosomal location Figure 5(c). This will fetch cluster loci, cluster score, total number of piRNAs within the cluster, cluster strandedness, prevalence of these piRNAs in minus/plus strand, and the corresponding characteristic motif of the cluster in that location. The link on the motif navigates to the website (https://meme-suite.org/meme) where users can perform further study on the motif.
2. ***Search mRNAs/lncRNAs/Repeats within piRNA Clusters***: Users can check if piRNA clusters are overlapped with mRNA/lncRNA loci or with the repeat elements.
3. ***Search piRNA Clusters in Syntenic Regions***: Users can search for piRNA clusters overlapping with syntenic regions by selecting a particular chromosome for both target and query organisms.

(c) Browse **‘piRNA Expression’**

1. ***Search Tissue specific expression***: User can view piRNA expression pattern by selecting tissue type and will be able to see the top 200 most abundantly expressed piRNAs by submitting the view option corresponding to the dataset.
2. ***Search Disease specific expression***: User can also retrieve same information as above for different disease systems.

We have provided an additional option of downloading the entire set of tissue/disease wise piRNA expression information for all the samples from the download section.

**Figure 6.**
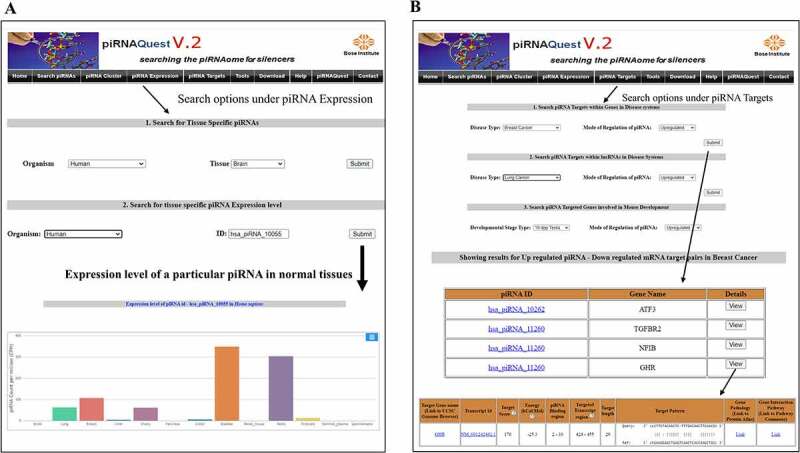
Web interfaces for easy access of piRNAQuest V.2 showing: (a) tissue wise expression values of individual piRNAQuest IDs and the corresponding output and (b) search options and detailed output for piRNA target prediction.

An additional search option is there under both the above mentioned menus to retrieve expression level of a particular piRNA in normal tissues of selected species or that in human disease systems Figure 6(a).

(d) Search ‘**piRNA Targets**’

1. ***Search Predicted Targets***: Users can search piRNA targets in mRNA/lncRNA for negatively correlated dataset by selecting the disease type/different developmental stages of mouse and the mode of regulation of the piRNA. After clicking the details option, user will be able to get the detailed prediction result and visualize the piRNA-target duplex structure Figure 6(b).
2. ***Search Curated Targets***: In this section, users can find literature curated piRNA-gene target pairs.

#### Tools

1. ***Dynamic piRNA cluster detection***: This tool can detect piRNA clusters where user can set parameters of their own, like the chromosomal coordinates, Eps distance and MinReads.
2. ***piRNA Target prediction***: Users can provide the piRNA and target sequences of their own choice along with their desired energy parameters and threshold target score to predict piRNA targets.
3. ***Ping-pong signature detection***: Users need to provide piRNA sequences in.fasta format to visualize ping-pong signature pattern within them.

## Supplementary Material

Supplemental Material

## Availability

piRNAQuest V.2 is available at http://dibresources.jcbose.ac.in/zhumur/pirnaquest2. Files can be freely downloaded and used in accordance with the GNU Public License.
