# Supplementary material for: piRNAQuest V.2: an updated resource for searching through the piRNAome of multiple species
Source: RNA Biol. 2021 Dec 29;19(1):12–25. doi: 10.1080/15476286.2021.2010960 (PMC8786328; doi:10.1080/15476286.2021.2010960)
Supplement: Supplemental Material [file KRNB_A_2010960_SM9256.zip › supplementary/ST1.docx]

**Supplementary Table 1:**

| **Species** | **Phylum/Class** | **Genome Build** | **No of unique piRNAs** |
| --- | --- | --- | --- |
| Human | Chordata/Mammalia | GRCh38/hg38 | 51509 |
| Mouse |  | GRCm38/mm10 | 2357673 |
| Rat |  | RGSC 6.0/rn6 | 190191 |
| Chinese hamster |  | CHOK1S_HZDv1/criGriChoV2 | 25626 |
| Cow |  | UMD_3.1.1/bosTau8 | 32147 |
| Pig |  | Sscrofa11.1/susScr11 | 94818 |
| Platypus |  | ASM227v2/ornAna2 | 1400 |
| Rabbit |  | Broad/oryCun2 | 161564 |
| Dog |  | CanFam3.1/canFam3 | 1036427 |
| Horse |  | Broad/equCab2 | 1461782 |
| Marmoset |  | WUGSC 3.2/calJac3 | 1068209 |
| Crab-eating macaque |  | Macaca_fascicularis_5.0/macFas5 | 5380 |
| Rhesus macaque |  | BCM Mmul_8.0.1/rheMac8 | 55341 |
| Northern Tree Shrew |  | Broad/tupBel1 | 32701 |
| Big Brown Bat |  | EptFus1.0 | 228382 |
| Zebrafish | Chordata/Actinopterygii | GRCz10/danRer10 | 154562 |
| Chicken | Chordata/Aves | Gallus_gallus-5.0/galGal5 | 118784 |
| *Xenopus laevis* | Chordata/Amphibia | Xenopus_laevis_v2/xenLae2 | 1386083 |
| *Xenopus tropicalis* |  | JGI 9.1/xenTro9 | 11466 |
| *C. elegans* | Nematoda/Chromadorea | WS220/ce11 | 15365 |
| *Drosophila melanogaster* | Euarthropoda/Insecta | BDGP Release 6 + ISO1 MT/dm6 | 250103 |
| *Drosophila erecta* |  | droEre1 | 12055 |
| *Drosophila yakuba* |  | WUGSC 7.1/droYak2 | 26892 |
| *Drosophila virilis* |  | droVir2 | 20192 |
| Aedes | Arthropoda/Insecta | Aedes_aegypti.AaegL3 | 253408 |
| Silkworm |  | Bombyx_mori.ASM15162v1 | 223085 |
| California sea hare | Mollusca/Gastropoda | Broad 2.0/aplCal1 | 221 |
| Starlet sea anemone | Cnidaria/Anthozoa | ASM20922v1 | 2323 |
