# Supplementary figures and images for: piRNAQuest V.2: an updated resource for searching through the piRNAome of multiple species
Source: RNA Biol. 2021 Dec 29;19(1):12–25. doi: 10.1080/15476286.2021.2010960 (PMC8786328; doi:10.1080/15476286.2021.2010960)

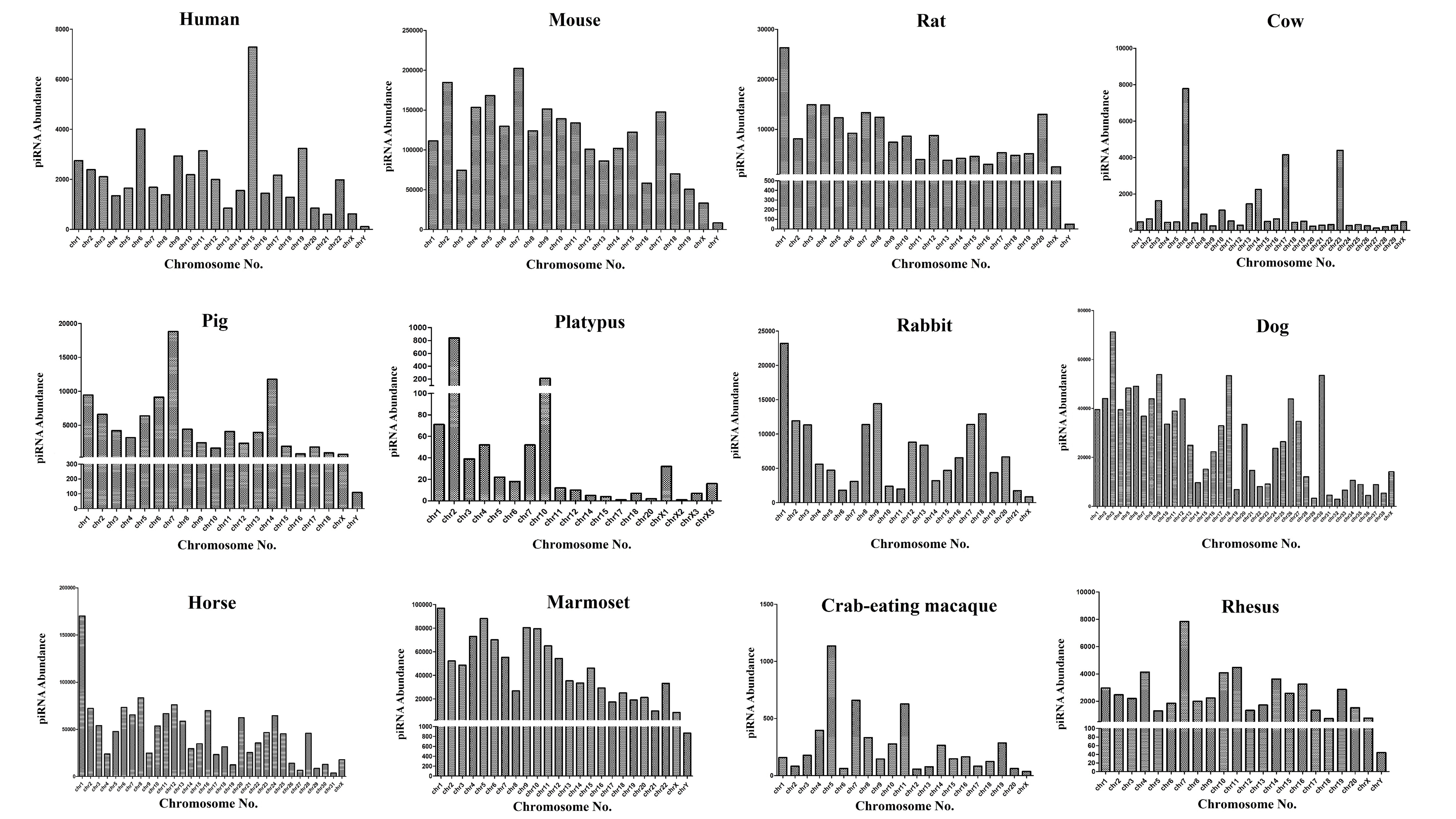

Supplement: Supplemental Material [file KRNB_A_2010960_SM9256.zip › supplementary/SF1.jpg]

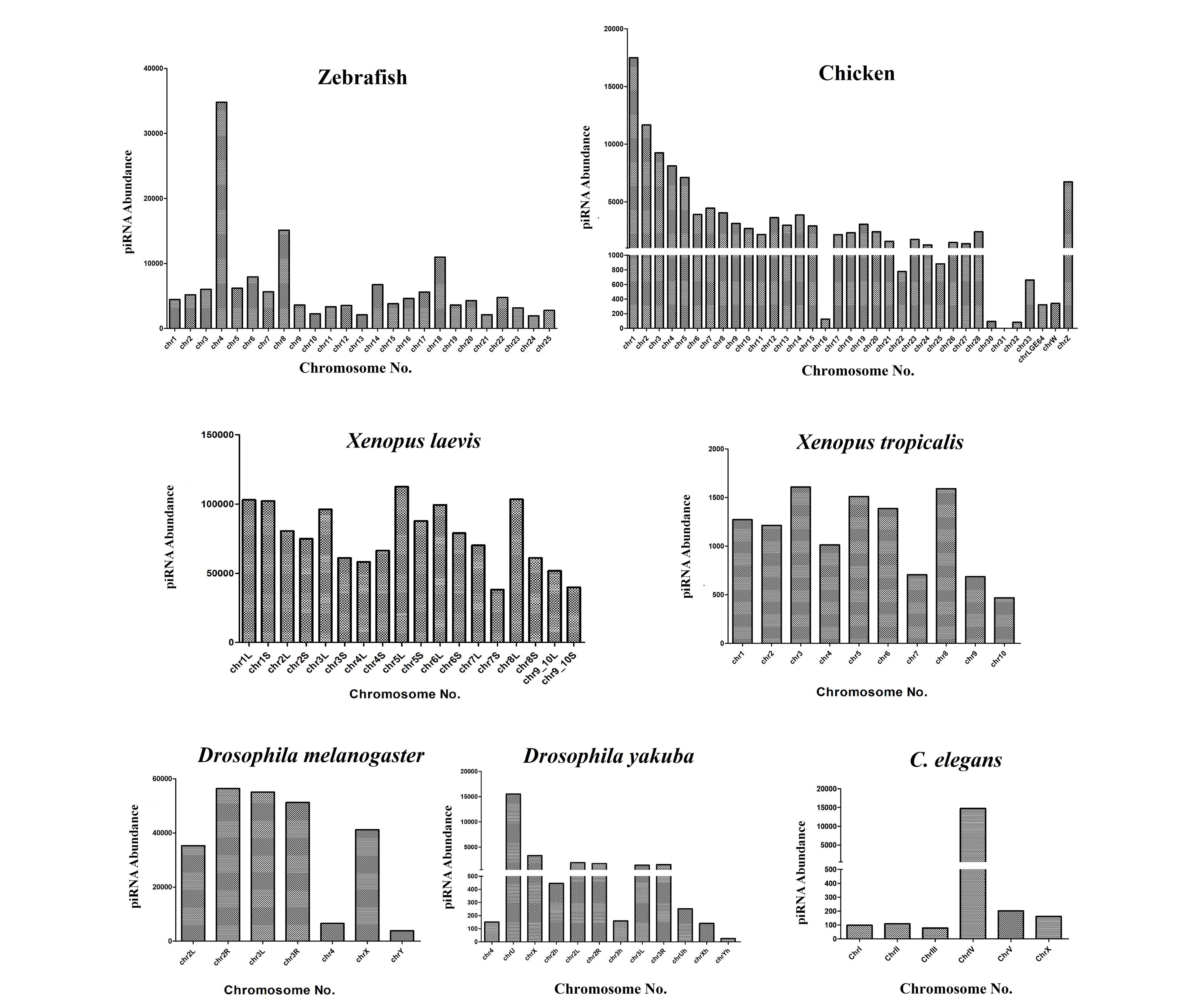

Supplement: Supplemental Material [file KRNB_A_2010960_SM9256.zip › supplementary/SF2.jpg]

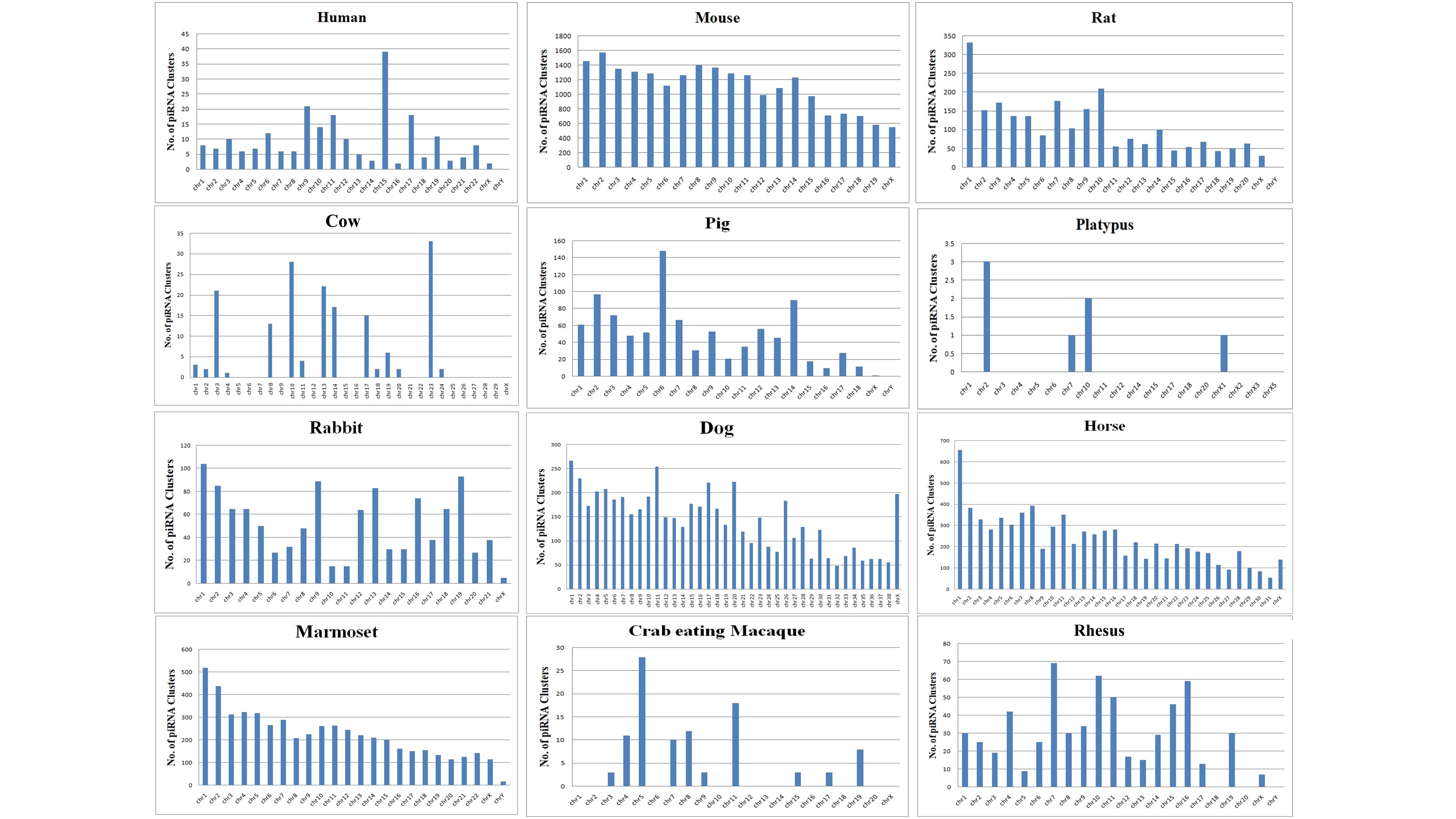

Supplement: Supplemental Material [file KRNB_A_2010960_SM9256.zip › supplementary/SF3.jpg]

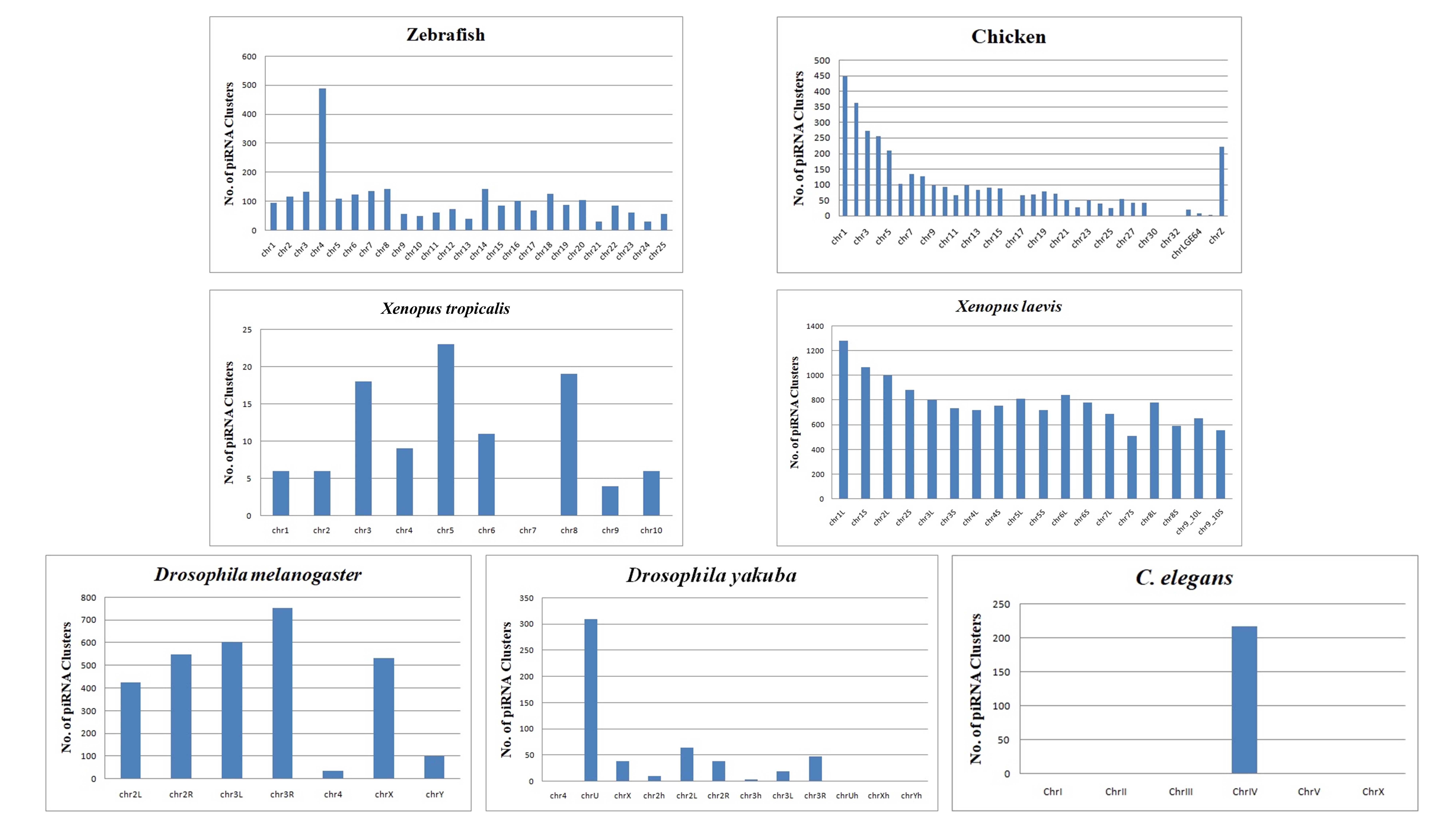

Supplement: Supplemental Material [file KRNB_A_2010960_SM9256.zip › supplementary/SF4.jpg]

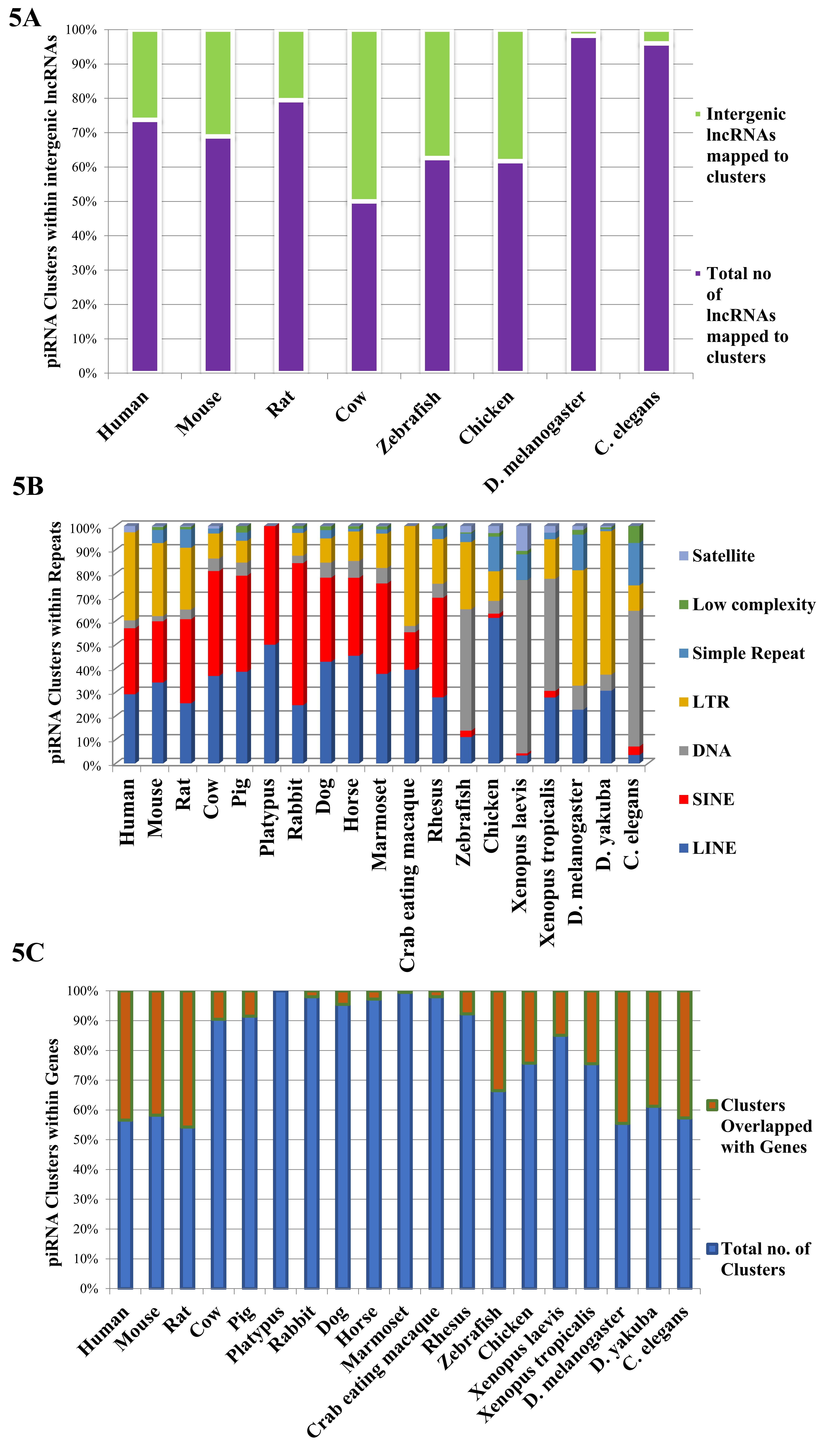

Supplement: Supplemental Material [file KRNB_A_2010960_SM9256.zip › supplementary/SF5.jpg]

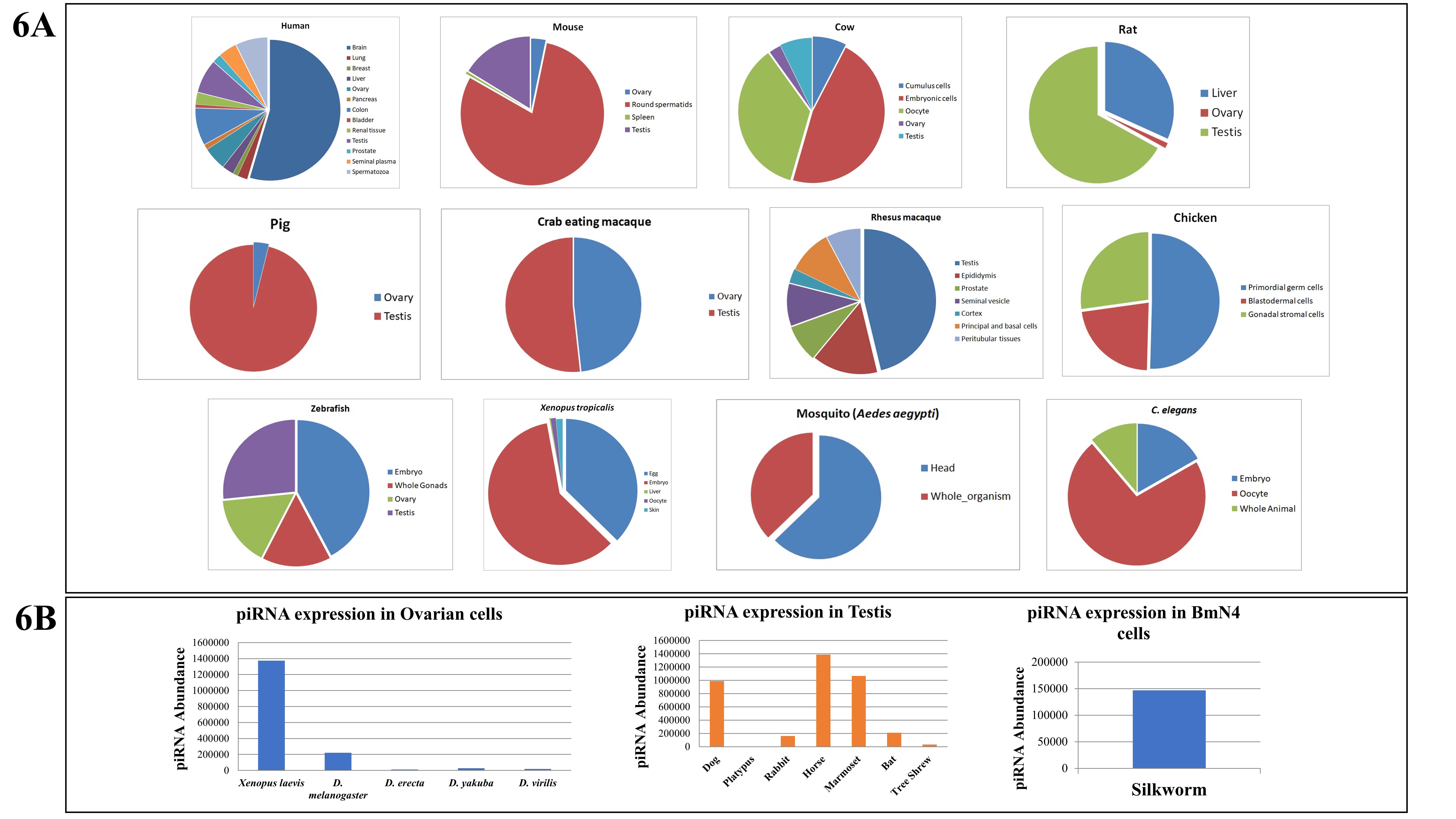

Supplement: Supplemental Material [file KRNB_A_2010960_SM9256.zip › supplementary/SF6.jpg]

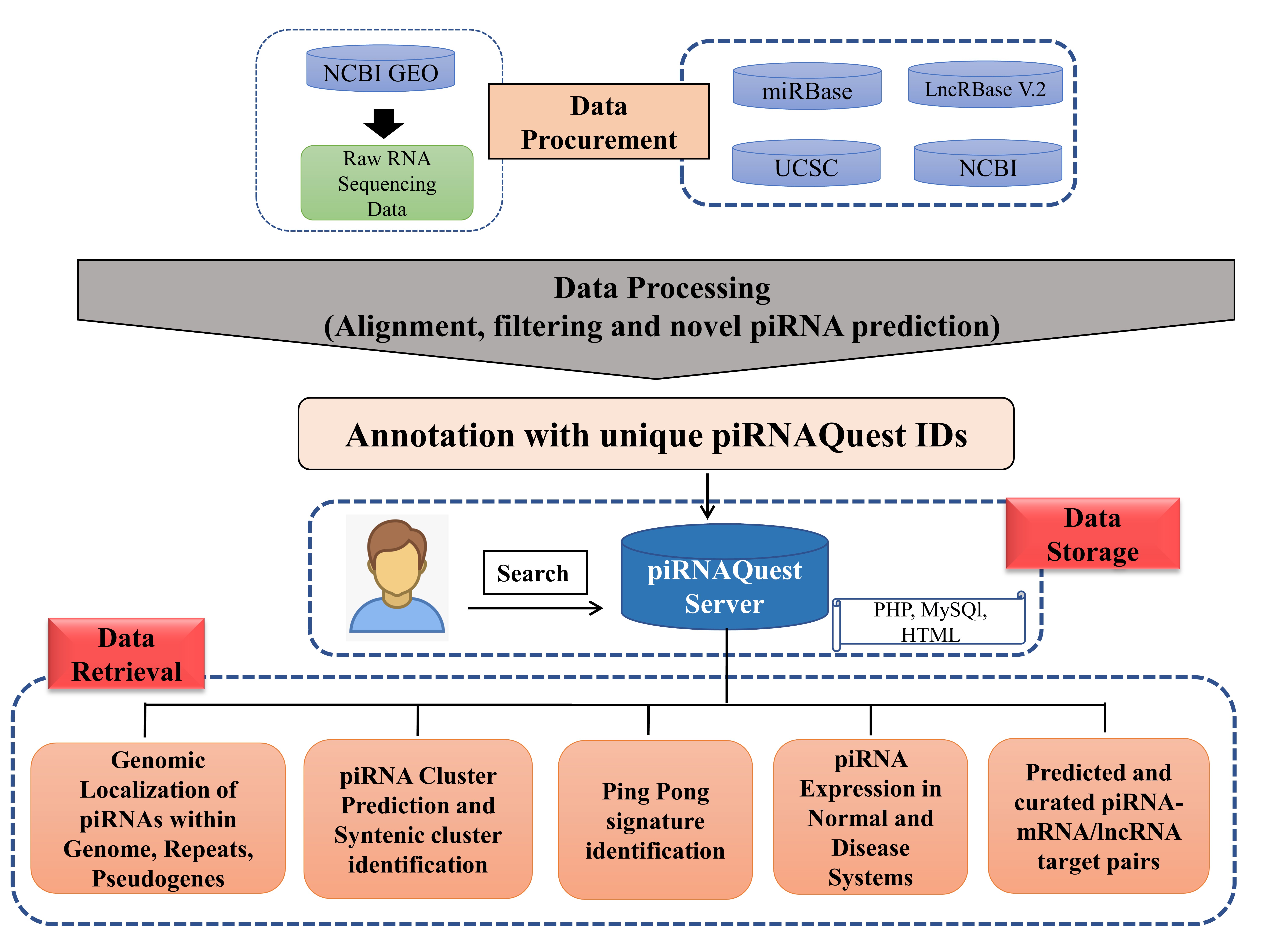

Supplement: Supplemental Material [file KRNB_A_2010960_SM9256.zip › supplementary/SF7.jpg]
