# Supplementary material for: piRNAQuest V.2: an updated resource for searching through the piRNAome of multiple species
Source: RNA Biol. 2021 Dec 29;19(1):12–25. doi: 10.1080/15476286.2021.2010960 (PMC8786328; doi:10.1080/15476286.2021.2010960)
Supplement: Supplemental Material [file KRNB_A_2010960_SM9256.zip › supplementary/Supplementary information.doc]

**Supplementary Figure and Table legends:**

**Supplementary Figure SF1:** Chromosomal distribution of piRNAs among mammals

**Supplementary Figure SF2:** Chromosomal distribution of piRNAs among other species

**Supplementary Figure SF3:** Chromosomal distribution of piRNA Clusters among mammals

**Supplementary Figure SF4:** Chromosomal distribution of piRNA Clusters among other species

**Supplementary Figure SF5:** Distribution of piRNA Clusters 3A. Within intergenic lncRNAs, 3B. Within Repeat elements and 3C. Within coding regions

**Supplementary Figure SF6:** piRNA expression of different species in A: Multiple tissues; B: Single tissues

**Supplementary Figure SF7:** Workflow of piRNAQuest V.2

**Supplementary Table ST1:** Genome builds information and number of piRNA entries corresponding to different species for piRNAQuest V.2

<http://dibresources.jcbose.ac.in/zhumur/pirnaqst2db/ST1.docx>

**Supplementary File S1:** Feature wise comparison of different piRNA database with piRNAQuest V.2

<http://dibresources.jcbose.ac.in/zhumur/pirnaqst2db/S1.xlsx>

**Supplementary File S2:** NCBI GEO data accession IDs for normal tissue specific expression analysis of piRNAs for all the species

<http://dibresources.jcbose.ac.in/zhumur/pirnaqst2db/S2.xlsx>

**Supplementary File S3:** NCBI GEO data accession IDs for disease specific expression analysis of piRNAs for all type of diseases

<http://dibresources.jcbose.ac.in/zhumur/pirnaqst2db/S3.xlsx>

**Supplementary File S4:** NCBI GEO data accession IDs for long RNAseq data used for predicting piRNA-target interactions

<http://dibresources.jcbose.ac.in/zhumur/pirnaqst2db/S4.xlsx>
